# External validation of a deep learning algorithm for automated echocardiographic strain measurements

**DOI:** 10.1093/ehjdh/ztad072

**Published:** 2023-11-20

**Authors:** Peder L Myhre, Chung-Lieh Hung, Matthew J Frost, Zhubo Jiang, Wouter Ouwerkerk, Kanako Teramoto, Sara Svedlund, Antti Saraste, Camilla Hage, Ru-San Tan, Lauren Beussink-Nelson, Maria L Fermer, Li-Ming Gan, Yoran M Hummel, Lars H Lund, Sanjiv J Shah, Carolyn S P Lam, Jasper Tromp

**Affiliations:** Division of Medicine, Akershus University Hospital, Lørenskog, Norway; K.G. Jebsen Center of Cardiac Biomarkers, University of Oslo, Oslo, Norway; Division of Cardiology, Department of Internal Medicine, MacKay Memorial Hospital, Taipei, Taiwan; Institute of Biomedical Sciences, MacKay Medical College, New Taipei, Taiwan; Us2.ai, Singapore, Singapore; Us2.ai, Singapore, Singapore; National Heart Centre Singapore, Singapore, Singapore; Department of Dermatology, Amsterdam Institute for Infection and Immunity, Cancer Centre Amsterdam, Amsterdam UMC, University of Amsterdam, Amsterdam, The Netherlands; Department of Biostatistics, National Cerebral and Cardiovascular Center, Osaka, Japan; Department of Clinical Physiology, Institute of Medicine, Sahlgrenska University Hospital, University of Gothenburg, Gothenburg, Sweden; Ribocure Pharmaceuticals AB/Ribo Life Science Co Ltd, Gothenburg, Sweden; Heart Center, Turku University Hospital, University of Turku, Turku, Finland; Department of Cardiology, Heart, Vascular and Neuro Theme, Karolinska University Hospital, Stockholm, Sweden; Department of Medicine, Cardiology Unit, Karolinska Institutet, Stockholm, Sweden; National Heart Centre Singapore, Singapore, Singapore; Division of Cardiology, Department of Medicine, Northwestern University Feinberg School of Medicine, Chicago, IL, USA; Early Clinical Development, Research and Early Development, Cardiovascular, Renal and Metabolism, BioPharmaceuticals R&D, AstraZeneca, Gothenburg, Sweden; Ribocure Pharmaceuticals AB/Ribo Life Science Co Ltd, Gothenburg, Sweden; Department of Molecular and Clinical Medicine, Institute of Medicine, Sahlgrenska Academy at the University of Gothenburg, Gothenburg, Sweden; Us2.ai, Singapore, Singapore; Department of Cardiology, Heart, Vascular and Neuro Theme, Karolinska University Hospital, Stockholm, Sweden; Division of Cardiology, Department of Medicine, Northwestern University Feinberg School of Medicine, Chicago, IL, USA; National Heart Centre Singapore, Singapore, Singapore; Duke-National University of Singapore Medical School, Singapore, Singapore; National Heart Centre Singapore, Singapore, Singapore; Duke-National University of Singapore Medical School, Singapore, Singapore; Saw Swee Hock School of Public Health, National University of Singapore, Singapore, Singapore

**Keywords:** Deep learning, Echocardiography, Strain, Global longitudinal strain, Heart failure, Artificial intelligence

## Abstract

**Aims:**

Echocardiographic strain imaging reflects myocardial deformation and is a sensitive measure of cardiac function and wall-motion abnormalities. Deep learning (DL) algorithms could automate the interpretation of echocardiographic strain imaging.

**Methods and results:**

We developed and trained an automated DL-based algorithm for left ventricular (LV) strain measurements in an internal dataset. Global longitudinal strain (GLS) was validated externally in (i) a *real-world* Taiwanese cohort of participants with and without heart failure (HF), (ii) a core-lab measured dataset from the multinational prevalence of microvascular dysfunction-HF and preserved ejection fraction (PROMIS-HFpEF) study, and regional strain in (iii) the HMC-QU-MI study of patients with suspected myocardial infarction. Outcomes included measures of agreement [bias, mean absolute difference (MAD), root-mean-squared-error (RMSE), and Pearson’s correlation (R)] and area under the curve (AUC) to identify HF and regional wall-motion abnormalities. The DL workflow successfully analysed 3741 (89%) studies in the Taiwanese cohort, 176 (96%) in PROMIS-HFpEF, and 158 (98%) in HMC-QU-MI. Automated GLS showed good agreement with manual measurements (mean ± SD): −18.9 ± 4.5% vs. −18.2 ± 4.4%, respectively, bias 0.68 ± 2.52%, MAD 2.0 ± 1.67, RMSE = 2.61, R = 0.84 in the Taiwanese cohort; and −15.4 ± 4.1% vs. −15.9 ± 3.6%, respectively, bias −0.65 ± 2.71%, MAD 2.19 ± 1.71, RMSE = 2.78, R = 0.76 in PROMIS-HFpEF. In the Taiwanese cohort, automated GLS accurately identified patients with HF (AUC = 0.89 for total HF and AUC = 0.98 for HF with reduced ejection fraction). In HMC-QU-MI, automated regional strain identified regional wall-motion abnormalities with an average AUC = 0.80.

**Conclusion:**

DL algorithms can interpret echocardiographic strain images with similar accuracy as conventional measurements. These results highlight the potential of DL algorithms to democratize the use of cardiac strain measurements and reduce time-spent and costs for echo labs globally.

## Graphical Abstract

**Graphical Abstract:**
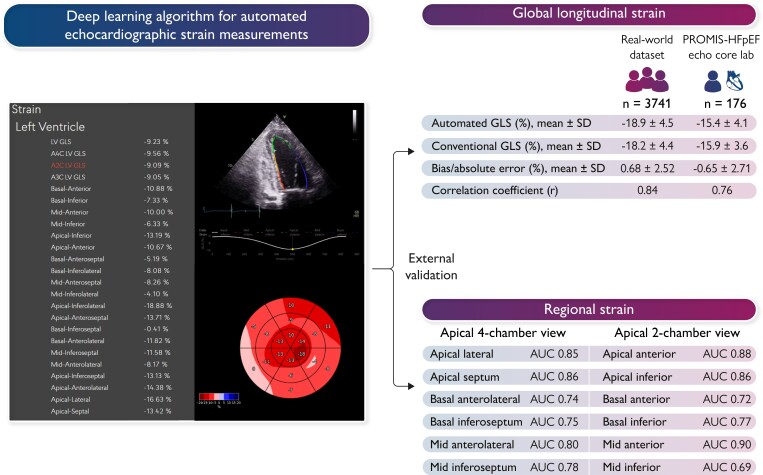

## Introduction

Strain imaging reflects myocardial deformation and is currently the most sensitive measure of left ventricular (LV) function. Reduced LV global longitudinal strain (GLS) predicts adverse clinical outcomes and helps in the early diagnosis of myocardial diseases, particularly heart failure (HF).^1^ GLS measurement is also crucial for monitoring subclinical cardiac disease progression, such as during cardiotoxic cancer treatment, where its use has a class 1 recommendation.^2^ Attempts have been made to standardize the measurement of myocardial strain^3^ and although current platforms include semi-automated strain measurements, the clinical application remains limited due to the expertise required to perform the measurement and high intra-observer variability.^4^ Fully automated strain algorithms have the potential to overcome these challenges.

Deep learning (DL) algorithms, such as convolutional neural networks (CNNs), can automate the interpretation of echocardiographic images. We previously demonstrated the development and validation of a DL workflow to assess cardiac volumes and Doppler measurements automatically.^5,6^ Previous attempts showed the potential of DL algorithms to automate LV GLS assessment.^7–10^ However, these attempts were limited by insufficient external validation, which is critical to determine the reproducibility and generalizability of the algorithm to new and different patients and settings. Moreover, no study has assessed the accuracy of a DL-based algorithm for echocardiographic regional strain measurements.

Therefore, we developed and trained a CNN DL-based vendor-independent algorithm to automatically measure LV strain from standard echocardiographic apical views in an internal training dataset. We validated GLS and regional strain measurements produced with the DL-based automated workflow in three external validation cohorts: (i) real-world study of patients with and without HF, (ii) core-lab ‘gold-standard’ annotated echocardiograms from patients with HF and preserved ejection fraction (HFpEF), and (iii) patients with suspected acute myocardial infarction (AMI).

## Methods

### Derivation of DL-based workflow

We previously reported the development of our DL-based workflow.^5^ The automated DL-based workflow was prototyped through a collaboration between research institutions in Singapore [Bioinformatics Institute, Institute of High-Performance Computing, and Institute for Infocomm Research of the Agency for Science, Technology and Research (A*StaR)] using data from the Asian Network for Translational Research and Cardiovascular Trials (ATTRaCT) program (https://www.a-star.edu.sg/attract). The workflow was trained and internally validated from conventional echocardiography examinations performed by experienced sonographers using commercially available equipment, as previously described.^5^ The development of the algorithm and reporting of the findings are adherent to the Proposed Requirements for Cardiovascular Imaging-Related Machine Learning Evaluation (PRIME) checklist.^11^ Because we wanted to analyse the algorithm’s performance across the spectrum of image quality and analytical settings, we validated the LV GLS measurements in a core-lab quality dataset [Prevalence of Microvascular Dysfunction (PROMIS)-HFpEF] and real-world clinical dataset (Mackay Memorial Hospital). To validate regional strain measurements, we included the Hamad Medical Corporation (HMC)- Qatar University (QU) dataset. This dataset has comprehensive information on regional wall motion abnormalities and is a publicly available dataset harmonious with transparency and data sharing in research.

### DL-based workflow for left ventricular strain

The DL-based workflow consists of two modules: (i) the conventional two-dimensional (2D) echo module and (ii) the strain module. Details of the 2D echo module are available elsewhere.^5^ Briefly, the 2D module initiates from categorizing echocardiographic digital imaging and communications in medicine (DICOM) files into either 2D videos or Doppler images using CNN-based imaging classifiers. Then, identified 2D video clips were classified into either A4C, A2C, or A3C views. After automatically identifying and excluding low-quality images, high-quality 2D video clips are parsed to a CNN model, which traces the endocardial border of the LV for each frame in A4C, A2C, and A3C views. Cardiac cycles (starting from end-diastole and ending at end-systole) are identified based on the video-level volume curves with confirmation by an accompanying electrocardiogram, if available. Linear measurements and chamber volumes were made using respective annotated views. The 2D module of the DL-based workflow has been demonstrated to categorize and classify 2D video and Doppler modalities with an accuracy ranging from 0.91 to 0.99 and was able to automatically annotate LV volumes from A2C and A4C views with mean Dice coefficients (measures of accuracy) of 93.0% to 93.8%, respectively.^5^

**Figure 1:**
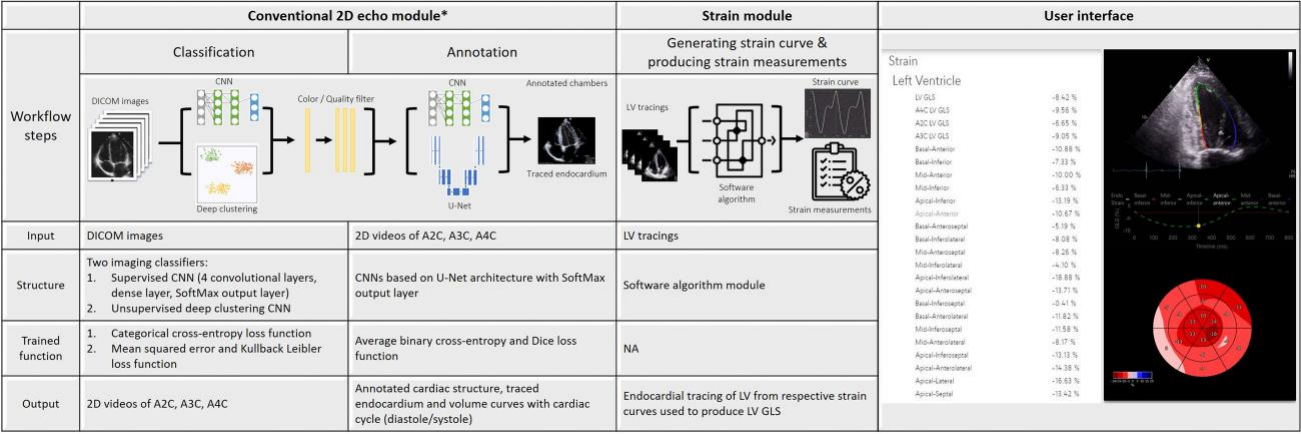
Deep learning-based workflow for automated global longitudinal strain measurement. *Conventional two-dimensional echo module was inherited from our previous work which includes tracing of left ventricular endocardial border and identification of cardiac cycle (diastole and systole) from annotated left ventricular volumes.

Using the annotated and endocardium-traced video clips of LV produced in the conventional 2D echo module, circumferential lengths of a traced endocardium for each frame were measured and were projected as drift corrected strain curves based on the cardiac cycle identified by video-level volume curves (*Figure 1*). This strain module is own developed and based on speckle tracking by a generic Optical Flow algorithm (see Supplementary material online, *Video***)**. The algorithm automatically prioritize LV focused apical views. If apical views were not available in the LV focused format, the algorithm will use regular A4C, A2C, and A3C views. The end-diastole LV annotations were tracked throughout the cardiac cycle using optical flow tracking. The annotations on each frame were used to guide and stabilize the tracked annotation. GLS was defined as the percentage change in the length of traced endocardium from end-diastole to end-systole. The algorithm does not average all available videos from the echocardiographic examination but selects the video with the highest quality view based on the view classifier confidence to maximize the likelihood of the measurements being correct. The final GLS value is presented as the average of all cycles on that video. The total LV GLS was estimated as the average of those determined from A4C, A3C, and A2C views.

### Quality assurance

To maintain the quality of automated GLS measurements, sequential quality checks were applied to identify and classify videos and images, as well as to produce automated measurements (see Supplementary material online, *Figures S1* and *S2*). Sequential quality checks included both components of imaging and measurement quality. The imaging quality was ascertained based on the highest probability produced by the view classifier (CNN’s SoftMax layer) to identify the highest quality loop for each individual. The measurement quality was evaluated based on confidence checks for heart rate, displacement of the region of interest (ROI) annotation trace, and congruency between systolic and diastolic phased with the electrocardiogram.

### External validation of GLS in a real-world cohort

The echocardiography examinations were performed by experienced local sonographers or cardiologists using the Vivid i system (GE Healthcare, Little Chalfont, UK) at the Mackay Memorial Hospital, Taipei, Taiwan, during annual cardiovascular health check-up and at outpatient clinic from June 2009 to December 2012.^12^ Images were analysed offline using endocardial border manual tracing by the same experienced technician, using proprietary software (EchoPAC version 10.8). The mean frame rate was between 60 and 80 frames/sec and LV GLS was averaged from three individual LV apical views (A2C, A4C, and A3C) based on the semi-automated speckle-tracking algorithm. Participants were excluded if they had congenital heart disease, significant valvular disease, isolated right-sided HF, pulmonary hypertension, acute coronary syndrome, or end-stage renal disease (estimated glomerular filtration rate <15 mL/min/1.73m^2^). HF diagnosis was independently adjudicated by an experienced HF cardiologist based on signs, symptoms, biomarkers and cardiac imaging, and classified as either HF with reduced EF (HFrEF), HFpEF, or hypertensive heart disease without HF. Demographics and clinical characteristics of the participants are presented in Supplementary material online, *Table S1*. Data was retrospectively identified, and a waiver of consent was obtained from the Mackay Memorial Hospital institutional review board.

### External validation of GLS in HFpEF

Design and details about the PROMIS-HFpEF study have been described elsewhere.^13^ In short, PROMIS-HFpEF was a prospective multinational, multi-centre observational study designed to identify the prevalence and correlates of coronary microvascular reserve in patients with HFpEF. Demographics and clinical characteristics of the analysed patients from the PROMIS-HFpEF are presented in Supplementary material online, *Table S2*. All patients underwent conventional two-dimensional (2D) echocardiography including LV strain measurements by speckle-tracking between November 2013 and May 2017.^13^ All echocardiographic measurements were performed by experienced research sonographers using GE EchoPAC echocardiographic analysis software (GE Healthcare, Wakaesha, WI) at a single core lab located at Northwestern University Echocardiography Core Laboratory. These measurements were further verified by investigators with expertise in echocardiography. All participants gave written informed consent, and the study complies with the Declaration of Helsinki which was reviewed and approved by the institutional review board at each participating site.

### External validation of regional strain in patients with suspected acute myocardial infarction

The HMC-QU dataset consists of a collection of A4C and A2C 2D echocardiography recordings (Philips or GE Vivid) of patients with suspected AMI, that is publicly available to the research community, as previously described.^14,15^ All patients had had no history of previous AMI and echocardiography recordings were obtained within 24 h of hospital admission and before coronary angiography. Regional wall-motion abnormalities included hypokinesia and akinesia and were classified for each myocardial segment by cardiologists at the HMC Hospital. Myocardial segments were analysed as recommended for assessing wall motion and regional strain, excluding the apical cap.^1^ Six segments were identified from A4C view; basal-inferoseptal, mid-inferoseptal, apical-septal, apical-lateral, mid-anterolateral, basal-anterolateral and six segments in A2C view; basal-inferior, mid-inferior, apical-inferior, apical-anterior, mid-anterior, and basal-anterior. In total, the HMC-QU dataset comprises 162 A4C view recordings (93 patients with acute AMI and 69 patients without AMI) and 130 A2C view recording (68 patients with acute AMI and 62 patients without AMI). All available images were included in this analysis. The data was been approved by the local ethics board of the HMC hospital in February 2019.

### Statistical methods

Numerical data with normal distribution are presented with mean ± standard deviation (SD) and non-normally distributed variables are presented as median (Q1, Q3). Mean absolute difference (MAD), root-mean-squared error (RMSE), and Pearson’s correlation coefficients (r) were estimated to assess the performance of the automated strain measurements against manual measurements. Agreement between the automated and manual GLS measurements was assessed by Bland-Altman analysis, with an estimated mean bias (the mean of the manual measurement minus automated measurement) and its lower and upper confidence limits.^16^ The normality of the bias was tested by the Shapiro-Wilk test. The ability to discriminate between patients with and without HF (HFpEF and HFrEF) for GLS and the ability to identify regional wall-motion abnormalities for regional strain was calculated by receiver operating characteristics area under the curve (AUC) for each segment.

Python version 3.8 was used to develop the DL-based workflow. Bland-Altman analyses were performed with Python API. Other cross-sectional analyses including classification performance were performed with STATA 15.0 (College Station, TX). A two-tailed *P*-value of <0.05 was considered statistically significant.

## Results

### External validation GLS in a real-world cohort

External validation of the automated strain measurements in a *real-world* cohort was performed in 4228 individuals with conventional GLS from a community-based cohort. Participants were aged mean 55 ± 15 years, 63% were men, 9% had atrial fibrillation (AF) and 16% had established coronary artery disease (CAD) (see Supplementary material online, *Table S1*). HF was present in 937 individuals [537 HFpEF and 400 HF with reduced EF (HFrEF)] and 428 had hypertensive heart disease without HF. The DL workflow successfully analysed apical 4-chamber [A4C], apical 2-chamber [A2C], and apical 3-chamber [A3C] GLS in 3741 out of 4228 studies (89%) in the *real-world* external validation set. Supplementary material online, *Figure S1* presents the number of echocardiograms passed in each step of sequential quality checks for LV GLS. The algorithm excluded 164 of the 4228 studies (3.9%) due to failure in view identification of either A4C (*n* = 70), A2C (*n* = 64) or A2C (*n* = 30) views, 77 studies (1.8%) were excluded due to poor confidence in produced measurements of LV GLS (*n* = 18 for A4C, *n* = 35 for A2C and *n* = 24 for A3C), 59 (1.3%) studies failed the heart rate confidence check, 114 (2.7%) failed the displacement check, 51 (1.2%) failed the check for identification of systolic and diastolic frame and 22 (0.5%) were excluded for other reasons.

**Figure 2:**
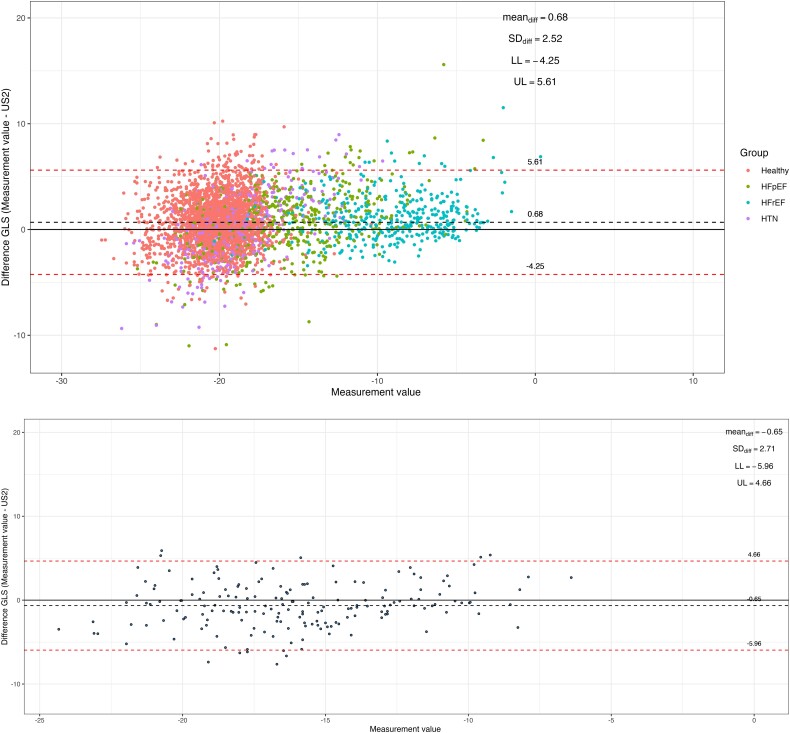
Bland-Altman plots for global longitudinal strain in prevalence of microvascular dysfunction-heart failure and preserved ejection fraction and a *real-world* dataset. Difference in total left ventricular global longitudinal strain (average of A4C, A2C, and A3C view) between automated and conventional measurements in a *real-world* cohort [top panel, *n* = 3741 stratified as healthy (*n* = 2376), hypertensive (*n* = 428), heart failure and preserved ejection fraction (*n* = 537) and heart failure with reduced ejection fraction (*n* = 400)] and in prevalence of microvascular dysfunction-heart failure and preserved ejection fraction (bottom panel, *n* = 176). *Abbreviations: GLS, global longitudinal strain; SD, standard deviation; LL, lower limit; UL, upper limit*.

The automated LV GLS measurements showed good agreement with the conventional strain measurements [r = 0.84, mean absolute difference (MAD) 2.0 ± 1.57, root-mean-squared-error (RMSE) 2.6]. The mean ± SD was −18.2 ± 4.4% for conventional GLS and −18.9 ± 4.5% for automated GLS measurements, yielding an average bias of 0.68 ± 2.52% (*Table 1*; *Figure 2*). The findings were consistent for separate analyses of A4C, A2C, and A3C views (see Supplementary material online, *Figure S3*). There was no difference in correlation between sinus rhythm (SR; r = 0.81 and mean bias 0.73%) and AF (r = 0.84 and mean bias 0.17), P_interaction_ = 0.15. The automated GLS successfully classified impaired manually measured GLS (using a threshold of −16%) with an AUC of 0.95 (95%CI 0.94–0.96) (see Supplementary material online, *Figure S4*).

**Table 1:** Comparison between automated and conventional global longitudinal strain measurements in a *real-world* dataset and in the Prevalence of Microvascular Dysfunction-heart failure and preserved ejection fraction study

	Real-world dataset	PROMIS-HFpEF
	*n* = 3741	*n* = 176
Automated GLS (%), mean ± SD	−18.9 ± 4.5	−15.4 ± 4.1
Conventional GLS (%), mean ± SD	−18.2 ± 4.4	−15.9 ± 3.6
Bias/absolute error (%), mean ± SD	0.68 ± 2.52	−0.65 ± 2.71
Root-mean-squared error	2.61	2.78
Mean absolute difference	2.0 ± 1.67	2.19 ± 1.71
Correlation coefficient (r)	0.84	0.76

Abbreviations: GLS, global longitudinal strain; LV, left ventricle; SD, standard deviation.

**Figure 3:**
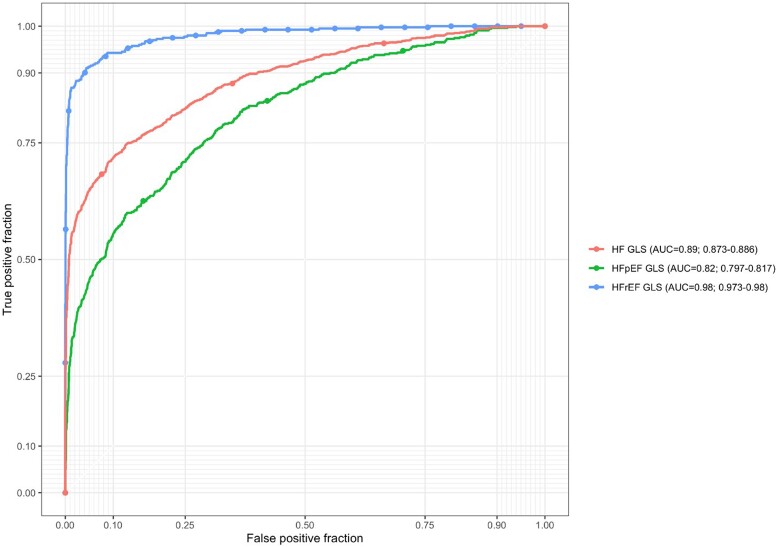
Receiver operating characteristic curves for classifying heart failure cases by automated global longitudinal strain in a *real-world* dataset. Separate curves for classifying heart failure with reduced ejection fraction, heart failure with preserved ejection fraction, and total heart failure compared to non-heart failure.

The performance [area under the receiver operating curve (AUC)] of the automated GLS in identifying HFrEF from non-HF was 0.98 (95%CI 0.97–0.98), HFpEF from non-HF was 0.82 (95%CI 0.80–0.82) and total HF from non-HF was 0.89 (95%CI 0.87–0.89) (*Figure 3*).

### External validation GLS in HFpEF

External validation of automated strain measurements in patients with HFpEF was performed against conventional measurements in 183 studies from the Prevalence of Microvascular Dysfunction (PROMIS)-HFpEF study. Patients had a mean age 74 ± 9 years, 56% were men, 53% had AF and 16% had established CAD (see Supplementary material online, *Table S2*). The DL workflow successfully analysed A4C, A2C, and A3C GLS in 176 out of 183 studies (96%) in the external validation set from PROMIS-HFpEF. Supplementary material online, *Figure S2* presents the number of echocardiograms passed in each step of sequential quality checks for LV GLS. The algorithm excluded one study from the analysis due to failure in view identification of either A4C, A3C, or A2C views and one study due to poor confidence in produced measurements of LV GLS. Additionally, five studies were excluded as they failed to pass the quality check for other reasons.

The automated LV GLS measurements showed good agreement with the strain measurements from the echo core lab (r = 0.76, MAD 2.19 ± 1.71, RMSE 2.78). The mean ± SD was −15.9 ± 3.6% for conventional GLS and −15.4 ± 4.1% for automated GLS measurements, yielding an average bias of −0.65 ± 2.71% (*Table 1*; *Figure 2*). The findings were consistent for separate analyses of A4C, A2C, and A3C views (see Supplementary material online, *Figure S5*).

### External validation cohort for regional strain

**Figure 4:**
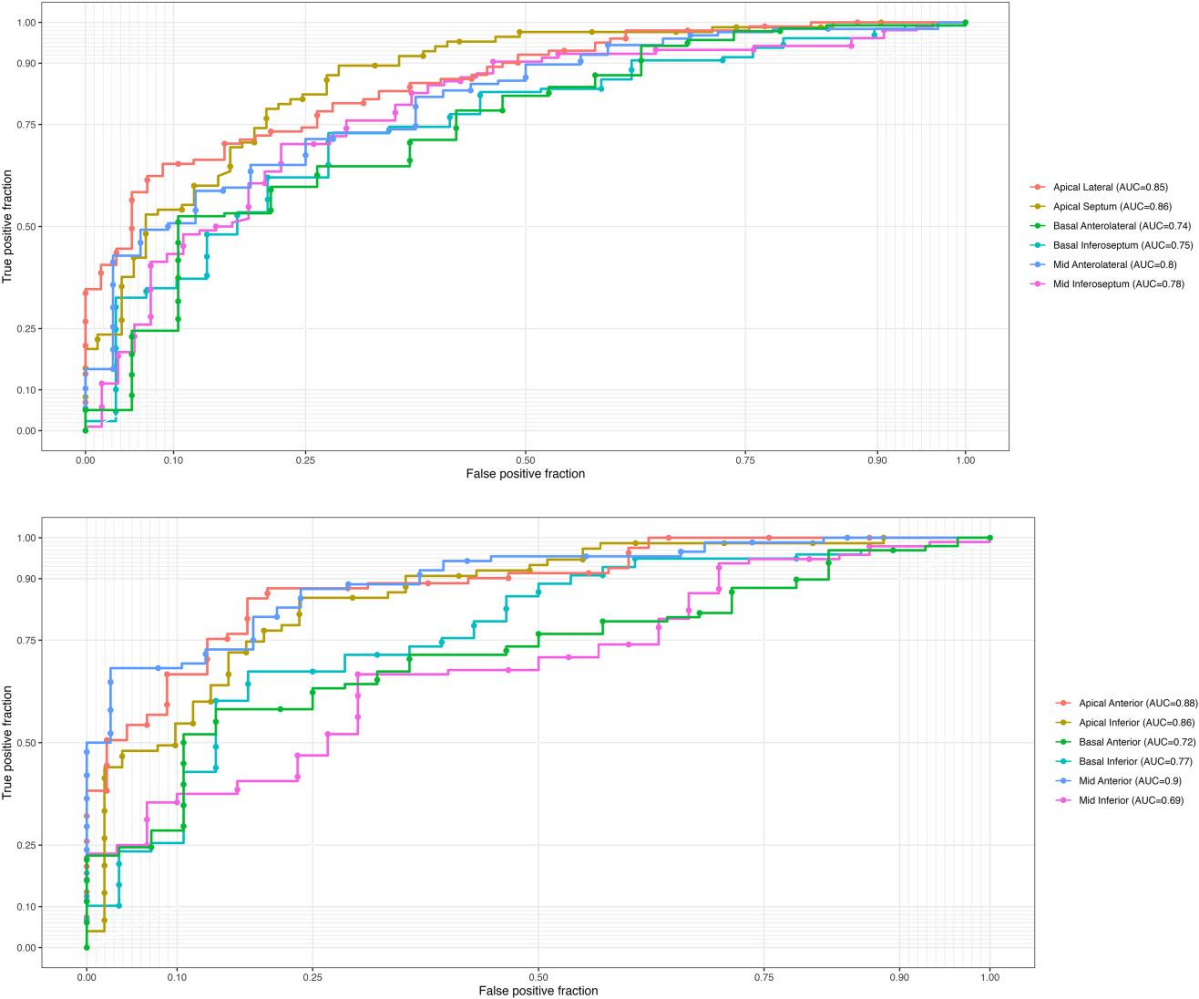
ROC curve for classifying regional wall-motion abnormalities by regional strain in patients with suspected AMI. Top panel represents six segments in apical 4-chamber view and the bottom panel six segments in apical 2-chamber view.

Patients with suspected AMI who had echocardiograms collected from 2018 to 2019 at HMC in Doha, Qatar in collaboration with researchers from Qatar University (QU) and Tampere University, Finland, were used for external validation of regional strain. Among 162 patients with suspected AMI who had available A4C view images, the DL workflow successfully analysed each LV segment in 156–158 (96–98%) studies. Among 130 patients with available A2C view, the DL workflow successfully analysed each LV segment in 125–126 (96–97%) studies. Automated regional strain identified regional wall-motion abnormalities in patients with suspected AMI with an AUC for each LV segment ranging from 0.74 to 0.86 in A4C view and from 0.69 to 0.90 in A2C view (average 0.80) (*Figure 4*).

## Discussion

We demonstrated good agreement between DL-based automated and conventional interpretation of echocardiography strain measurements. Our fully automated LV GLS measurements were highly correlated with conventional measurements made with commercially available software with a speckle-tracking approach in external *real-world* and core-lab measured datasets. We further demonstrated robust discriminatory performance of automated regional strain measurements for the detection of regional wall-motion abnormalities in patients with suspected AMI. Together, our results suggest that DL algorithms can aid in ‘democratizing’ the use of strain measurements in settings where resources or expertise are limited and even in clinically challenging circumstances of diagnosis of HFpEF and AMI.

We were able to produce automated GLS for 89% in the *real-world* dataset and 96% in the core lab dataset from PROMIS-HFpEF. The difference in the yields of GLS measurements was primarily related to poor image quality in a larger proportion of images in the *real-world* dataset. The main reasons for images to be excluded by the DL-based algorithm in the *real-world* dataset was failure to detect apical views (3.9%) and failure to pass the confidence check for displacement (mismatch in the ROI annotation between the systole and diastole; 2.7%). The number of studies excluded in PROMIS-HFpEF was limited, likely because PROMIS-HFpEF used a single standardized protocol for image acquisition and had higher-quality images. A *real-world* setting more likely introduces variation in imaging quality compared to prospective cohort studies, such as PROMIS-HFpEF. Importantly, the consistency in correlation and bias for the artificial intelligence (AI) algorithms between these very different cohorts used as ground truth increases the external validity of the findings.

Current commercially available software packages use semi-automated algorithms to estimate GLS.^17,18^ However, these existing algorithms still require considerable human inputs, i.e. detection of the myocardial border, editing the ROI width, etc. which is time consuming and introduces intra-observer variability. Deep learning can fully automate the interpretation of echocardiographic images.,^7–9,19–22^ including GLS. Two previous studies have compared the automated interpretation of strain imaging to conventional measurements. Both studies demonstrated good agreement between DL-based automated and conventional GLS measurements.^7,8^ For example, Salte et al. found a mean bias of only 1.4% in their internal validation cohort. This group has also demonstrated reduced test-retest variability in repeated echocardiograms for GLS analysis by AI as compared to manual measurements.^10^ Importantly, these studies did not externally validate their algorithms, which is a critical step before clinical implementation. Our results extend on these previous analyses by including two large *external* validation cohorts for GLS and demonstrating the feasibility of automated regional strain to identify patients with suspected AMI. In our study, the bias for GLS was less than 1% in both the *real-world* and core-lab read PROMIS-HFpEF study using echo core lab, demonstrating good agreement in datasets not used for training the algorithms.

To our knowledge, we are the first to validate automated GLS in patients with AF, as these were excluded from previous studies.^7–9^ We included participants irrespective of baseline rhythm, and our results show good agreement with manual measurements also in patients with AF. Automated GLS measurements in AF have the potential to substantially improve efficiency as averaged beat-to-beat measurements of GLS are immediately calculated. This is in contrast to averaging measurements of at least 5 cardiac cycles for each apical view as recommended for manually measured GLS.^1^ These are novel results that need to be further explored.

Automated GLS could accurately identify patients with HF from controls. Although GLS alone should not be used to diagnose HF, these data add to previous studies validating other key measures to diagnose HF, such as LV ejection fraction, left atrial volume, mitral flow, and mitral annular early diastolic velocity.^5,6^ The similar performance of automated and conventional GLS measurement for identifying patients with HF demonstrates the potential clinical utility of DL-based measurements. Furthermore, we were able to accurately detect regional wall-motion abnormalities in patients with suspected AMI using automated regional strain measurements. No previous studies have demonstrated these algorithms for echocardiography.

With the rapidly evolving field of AI-based echocardiography, it is soon expected to be part of clinical routine. Quick and accurate measurements of cardiac structure and function with low inter- and intra-reader variability allow for increased effectivity and availability of echocardiograms. In this study, we have demonstrated that an AI-based approach is not only reliable for measures of 2D video and Doppler images, but also accurately measures myocardial deformation in external validation cohorts. GLS is the most sensitive echocardiographic measure of systolic dysfunction and is ideal for assessing subtle changes in cardiac function. The new ESC Guidelines for cardio-oncology recommend (Class 1) GLS measurements in all patients with cancer having an echocardiogram.^2^ Furthermore, regional strain is also a sensitive measure of regional wall-motion abnormalities. By using traditional 2D-images, regional hypokinesis is difficult to visualize and prone to substantial inter-reader variability. Using automated regional strain measurements, we were able to accurately detect regional wall-motion abnormalities in patients with suspected AMI. Overall, fully automated strain has the potential to improve patient care for assessing diagnosis, risk stratification, and therapeutic monitoring.

There are few limitations in this study. The validation of our automated strain measurements was only tested against speckle-tracking approach measured by the same vendor produced software in both external cohorts. The lack of gold-standard approach in strain measurements, variability in manual strain measurements and inter-vendor variability may limit the generalizability of our results. In this study, the majority of images were acquired by GE equipment, however, the DL-algorithm is compatible with all the major manufacturers of echocardiography imaging.^23^ Finally, we were not able to relate our automated strain measurements to long-term outcomes.

To conclude, fully automated measurement of GLS and regional strain by DL-based workflow showed good agreement with conventional measurements in a core-lab-based cohort and in a *real-world* dataset. Automated measurement of GLS and regional strain correctly identified patients with HF and regional wall-motion abnormalities, respectively, with similar accuracy to conventional measurements. The application of DL-based measurements of GLS in real-world clinical settings and cost-benefit should be assessed in future studies.

## Supplementary material

Supplementary material is available at *European Heart Journal – Digital Health*.

## Supplementary Material

ztad072_Supplementary_Data

## Data Availability

Data from the HMC-QU-AMI study is publicly available. Data underlying this article from the MacKay cohort and PROMISE-HFpEF cannot be shared publicly due to the privacy of individuals which participated in the study.
