# Supplementary material for: External validation of a deep learning algorithm for automated echocardiographic strain measurements
Source: Eur Heart J Digit Health. 2023 Nov 20;5(1):60–8. doi: 10.1093/ehjdh/ztad072 (PMC10802824; doi:10.1093/ehjdh/ztad072)

### **Supplementary Material**

to

**External validation of a deep learning algorithm for automated echocardiographic strain measurements**

### **Supplementary Table 1.** Demographics and clinical characteristics of 4228 participants with available conventional GLS measurements in a real-world dataset from MacKay Memorial Hospital, Taiwan.

| **Demographics** |  |
| --- | --- |
| Age [years], mean (SD) | 55.1 (14.5) |
| Male, n (%) | 2657 (62.8%) |
| Asian ethnicity, n (%) | 4228 (100%) |
| BMI [kg/m^2^], mean (SD) | 25.1 (4.6) |
| **Comorbidities** |  |
| Hypertension, n (%) | 1468 (34.7%) |
| Atrial fibrillation, n (%) | 378 (8.9%) |
| Diabetes, n (%) | 785 (18.6%) |
| Coronary artery disease, n (%) | 669 (15.8%) |
| COPD, n (%) | 354 (8.4%) |
| eGFR [mL/min/1.73m^2^], mean (SD) | 79.7 (27.0%) |

### **Supplementary Table 2.** Demographics and clinical characteristics of 183 patients with available conventional GLS measurements in PROMIS-HFpEF.

| **Demographics** |  |
| --- | --- |
| Age [years], mean (SD) | 74.4 (8.7) |
| Women, n (%) | 81 (44%) |
| Race, n (%) |  |
| Asian | 165 (8%) |
| African American | 7 (4%) |
| White | 161 (88%) |
| Body mass index [kg/m^2^], mean (SD) | 29.7 (8.6) |
| **Comorbidities** |  |
| Heart failure, n (%) | 183 (100%) |
| Hypertension, n (%) | 153 (84%) |
| Atrial fibrillation, n (%) | 98 (54%) |
| Diabetes, n (%) | 52 (28%) |
| Coronary artery disease, n (%) | 29 (16%) |
| Chronic obstructive pulmonary disease, n (%) | NA |
| Creatinine [umol/L], median (Q1, Q3) | 93 (76, 115) |

**Suppl. Figure 1.** Flow chart of the number of echocardiograms passed in each step of sequential quality checks for LV GLS in the real-world general population dataset from MacKay Memorial Hospital

**Conventional LV GLS available**

**n = 4228**

Passed confidence check for systolic and diastolic frame probability

n = 3763 (89.0%)

Passed confidence check for displacement

n = 3814 (90.2%)

High confidence detection of apical views by AI algorithm

n = 3987 (94.2%)

Passed confidence check for heart rate

n = 3928 (92.9%)

Apical views detected by AI algorithm

n = 4064 (96.1%)

Low confidence A4C: n = 18

Low confidence A2C: n = 35

Low confidence A3C: n = 24

Failed heart rate confidence check: n = 59

Failed frame check A4C: n = 0

Failed frame check A2C: n = 21

Failed frame check A3C: n = 30

Discarded by shopping list: n = 22

Failed displacement check A4C: n = 8

Failed displacement check A2C: n = 45

Failed displacement check A3C: n = 61

Undetectable A4C: n = 70

Undetectable A2C: n = 64

Undetectable A3C: n = 30

**Successful automated GLS measurements**

**n = 3741 (88,5%)**

**Suppl. Figure 2.** Flow chart of the number of echocardiograms passed in each step of sequential quality checks for LV GLS in the PROMIS-HFpEF study

Discarded by shopping list: n = 1

Failed frame check A4C: n = 0

Failed frame check A2C: n = 0

Failed frame check A3C: n = 0

Failed displacement check A4C: n = 0

Failed displacement check A2C: n = 0

Failed displacement check A3C: n = 1

Low confidence A4C: n = 0

Low confidence A2C: n = 0

Low confidence A3C: n = 1

Undetectable A4C: n = 0

Undetectable A2C: n = 0

Undetectable A3C: n = 1

**Conventional LV GLS available**

**n = 183**

Failed heart rate confidence check: n = 3

**Successful automated GLS measurements**

**n = 176 (96.2%)**

Apical views detected by AI algorithm

n = 182 (99.5%)

High confidence detection of apical views by AI algorithm

n = 181 (98.9%)

Passed confidence check for heart rate

n = 178 (97.3%)

Passed confidence check for displacement

n = 177 (96.7%)

Passed confidence check for systolic and diastolic frame probability

n = 177 (96.7%)

**Suppl. Figure 3.** Bland-Altman plots for A4C, A2C and A3C view in a real-world general population cohort, stratified as HFrEF, HFpEF, hypertensive and healthy.


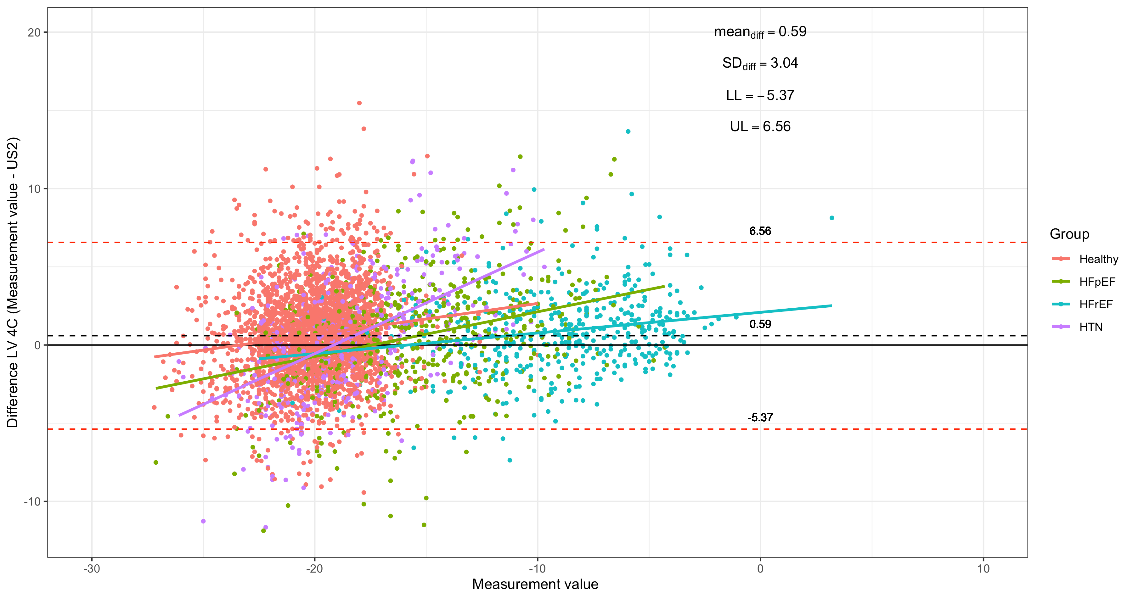

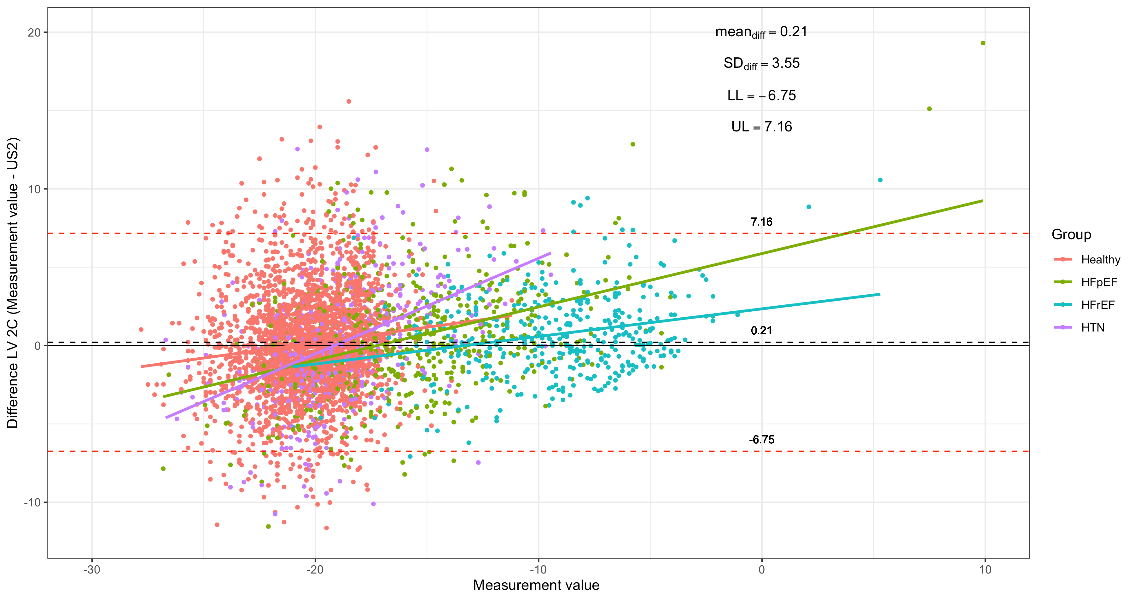

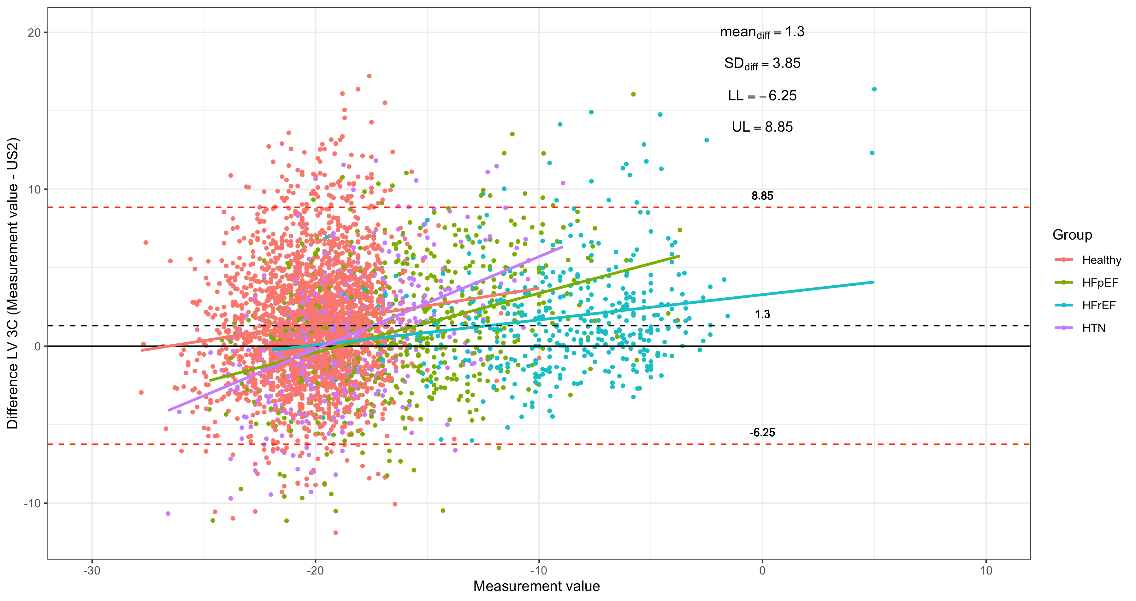


**Suppl. Figure 4.** Discriminatory performance of automated GLS for detecting impaired GLS (below -16%) in a real-world general population cohort


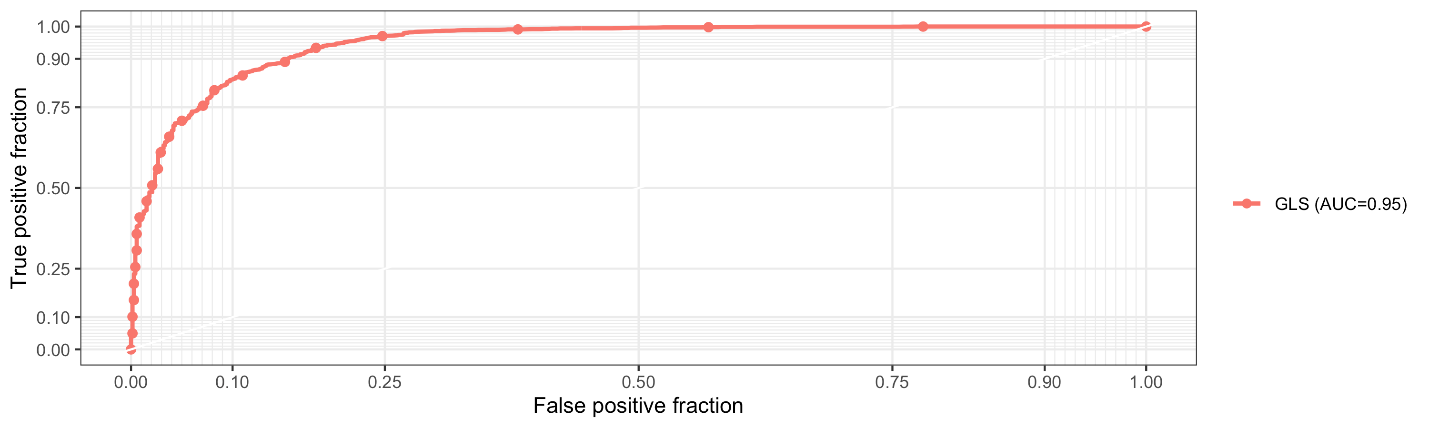


**Suppl. Figure 5** Bland-Altman plots for A4C, A2C and A3C view in PROMIS-HFpEF
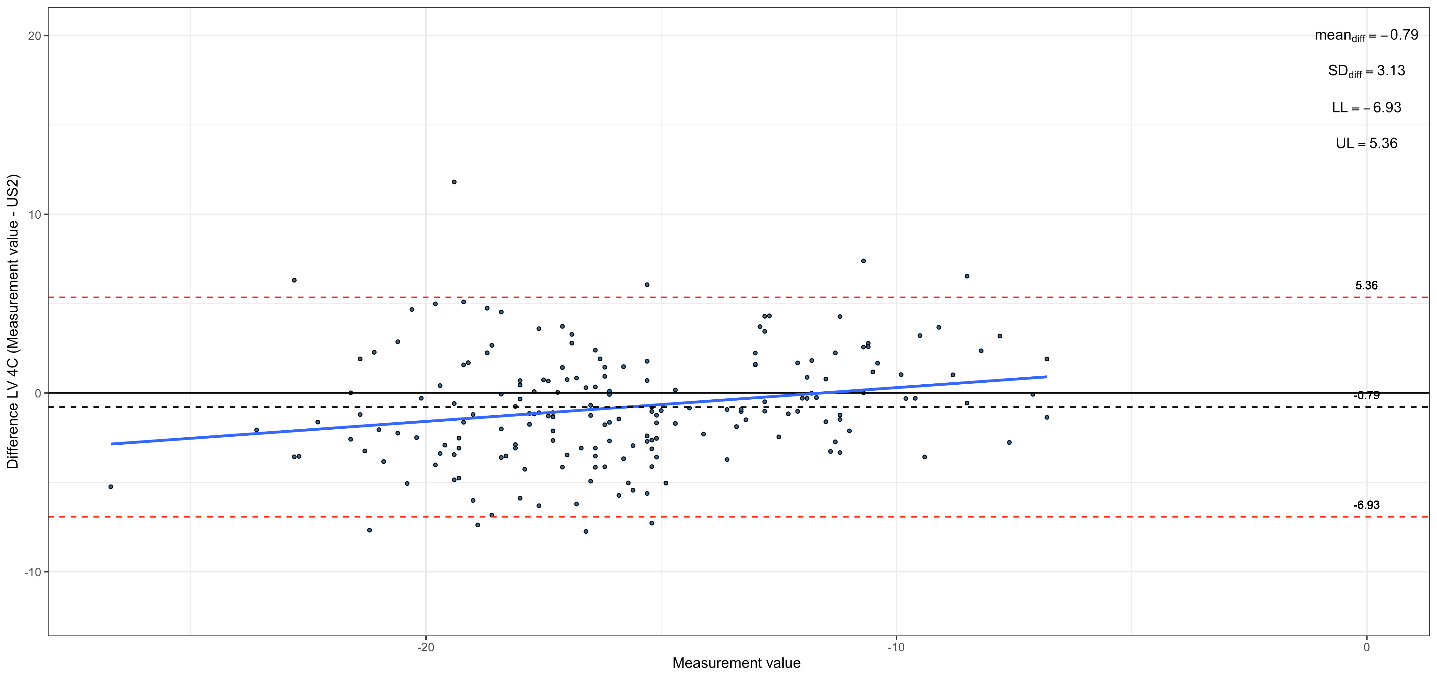


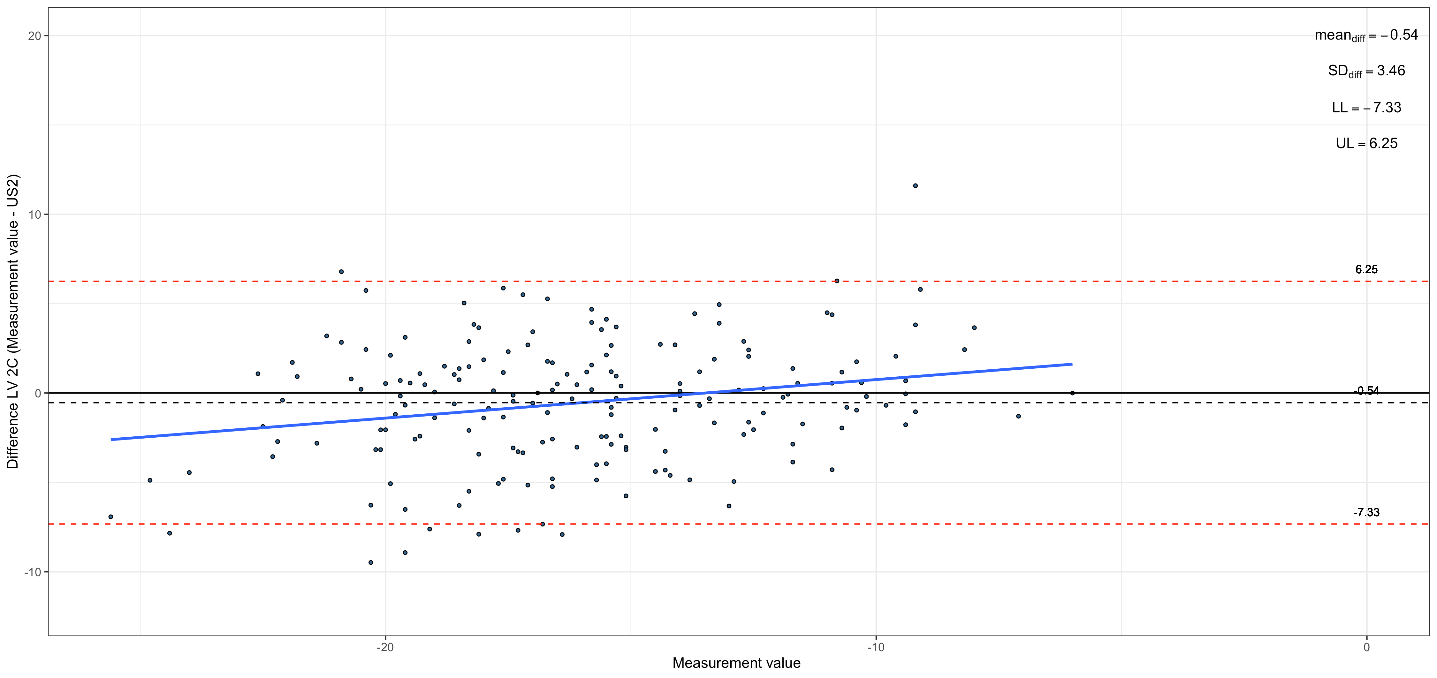

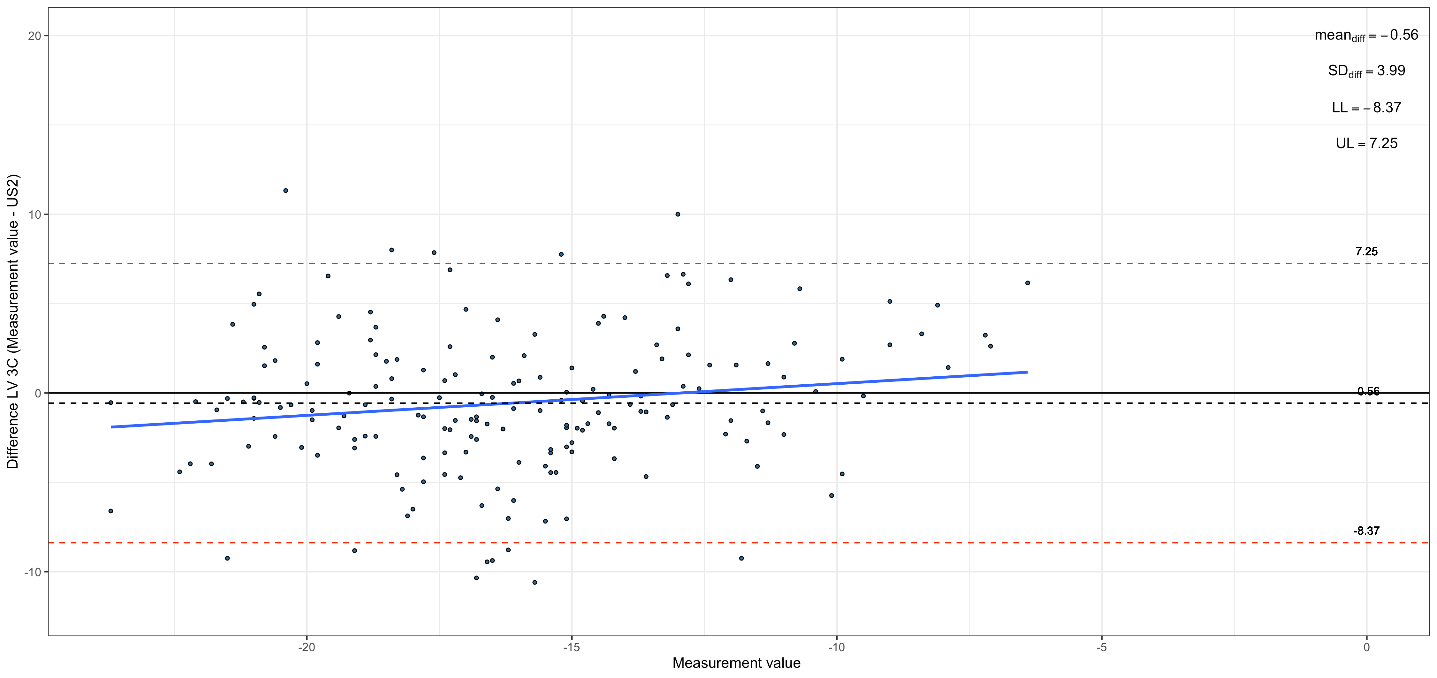

Supplement: ztad072_Supplementary_Data [file ztad072_supplementary_data.zip › AI strain Us2ai - Supplement EHJ Digital R1.docx]
